# Keratoconus International Consortium (KIC)- advancing keratoconus research

**DOI:** 10.1186/s12886-023-03087-w

**Published:** 2023-07-27

**Authors:** Srujana Sahebjada, Elsie Chan, Gerard Sutton, Chi Pui Calvin Pang, Srujana Sahebjada for KIC Members, Christopher Hodge, Elaine W. Chong, Faouzia Zemani-Fodil, Steve Wiffen, Grant Snibson, Nigel Morlet, Chameen Samarawickrama, John Males, Richard Mills, Peter Beckingsale, Kathryn Burdon, Vishal Jhanji, Pravin Krishna, Colin Chan, Abi Tenen, Athena Roufas, Tess Huynh, Rasik Vajpayee, Aanchal Gupta, Marcelo Reyes Silva, Mehran Zarei, Senthil Kumaran, Guofu Huang, Berthold Seitz, Shengwei Ren, Charles McGhee, Nigel Barker, Yves Kerdraon, Sundaram Natarajan, Wafaa Meteoukki, Andrea Ang, Mark Daniell, Paul N. Baird

**Affiliations:** 1 Centre for Eye Research Australia, Level 7, 32 Gisborne St, East Melbourne, Victoria 3002 Australia; 2 Department of Surgery, Ophthalmology, The University of Melbourne, Melbourne, Australia; 3 Royal Victorian Eye and Ear Hospital, Melbourne, Australia; 4 Sydney Medical School, The University of Sydney, Sydney, Australia; 5 NSW Tissue Banks, Sydney, Australia; 6 Chinese University of Hong Kong, Hong Kong, Hong Kong; 7 Envision Eye Centre, Sydney, Australia; 8 Aditya Jyot Eye Hospital, Mumbai, India; 9 Molecular and Cellular Genetics Laboratory, Oran University of Science and Technology - Mohamed Boudiaf (USTOMB), Oran, Algeria; 10 Lions Eye Institute, Perth, Australia

**Keywords:** Keratoconus, Consortium, Multi-centre project, Keratoconus progression and treatment

## Abstract

**Clinical relevance:**

The Keratoconus International Consortium (KIC) will allow better understanding of keratoconus.

**Background:**

Keratoconus is a disorder characterised by corneal elevation and thinning, leading to reduced vision. The current gaps in understanding of this disease will be discussed and the need for a multi-pronged and multi-centre engagement to enhance our understanding of keratoconus will be highlighted.

**Design:**

KIC has been established to address the gaps in our understanding of keratoconus with the aim of collecting baseline as well as longitudinal data on several fields.

**Participants:**

Keratoconus and control (no corneal condition) subjects from different sites globally will be recruited in the study.

**Methods:**

KIC collects data using an online, secure database, which enables standardised data collection at member sites. Data fields collected include medical history, clinical features, quality of life and economic burden questionnaires and possible genetic sample collection from patients of different ethnicities across different geographical locations.

**Results:**

There are currently 40 Australian and international clinics or hospital departments who have joined the KIC. Baseline data has so far been collected on 1130 keratoconus patients and indicates a median age of 29.70 years with 61% being male. A total of 15.3% report a positive family history of keratoconus and 57.7% self-report a history of frequent eye rubbing.

**Conclusion:**

The strength of this consortium is its international, collaborative design and use of a common data collection tool. Inclusion and analyses of cross-sectional and longitudinal data will help answer many questions that remain in keratoconus, including factors affecting progression and treatment outcomes.

## Introduction

Keratoconus is a progressive disorder characterised by abnormal corneal elevation, thickness distribution and corneal thinning [1]. It typically has an onset in the second decade of life and may progress until the fifth decade. It is a significant cause of visual impairment, especially in young adults. While diagnosis is unequivocal when clinical signs of corneal thinning and steepening are observed directly through biomicroscopy or corneal tomography, detection of early keratoconus remains challenging as there is currently no standard diagnostic or grading system for keratoconus. Further, there is no accepted definition of progression, and no ability to predict the prognosis and outcomes of treatment for individual patients [1–6].

The incidence of the disease is historically reported as 1 in 2000, based on longitudinal data collection from 1936 to 1982 from a single county in Minnesota, USA [7]. However, the limitations of this study, together with advances in imaging with corneal topography and tomography have suggested a higher incidence, including 1 in 375 being reported in the Netherlands based on national health insurance data [8]. Variations in prevalence across different populations are also apparent, with a prevalence of 1 in 42 reported from a college student population in Jerusalem, with a prevalence of 1.43% reported from a university student population in Syria [9] and a prevalence of 1 in 139 reported from a population-based study in Shahroud, Iran [10]. Evidence suggests a higher prevalence of keratoconus amongst Black and Hispanic communities in the USA and amongst Maori and Polynesian populations in New Zealand compared to Caucasian populations [11, 12]. Data from the United Kingdom has also shown Asian patients present at a younger age and with more advanced disease [13, 14].

The aetiology of keratoconus remains unknown, although it appears to be a complex, heterogeneous disorder with multiple causative factors that can be broadly classified as environmental, biomechanical, biochemical and genetic [1, 15–20]. The most likely mode of inheritance has been suggested as autosomal dominant although recessive genes may also exist [21–24]. An association with environmental and biomechanical factors is often described, with mechanical trauma from eye-rubbing, stimulation through contact lens wear or kerato-refractive laser surgery reported as contributing factors in the progression of keratoconus [25, 26]. The possible role of biochemical factors has also been implicated in the aetiology of this disease with elevated levels of matrix metalloproteinases and inflammatory cytokines found in keratoconus [27]. However, it is unclear if this is reflective of a cause or effect.

One of the main limitations of existing studies on keratoconus has been the relatively small number of patients that have been studied and reported, leading to underpowered analyses and false positive findings that limit generalisability of the results. Furthermore, differences in study design, patient populations, sampling methods, diagnostic methodology, outcome measurements, and length of follow-up have precluded the development of an overall predictive model for keratoconus. Diagnosis at the early stages of the disease is critical as corneal cross linking (CXL) can be performed to slow the progression of the condition to maintain vision and corneal regularity [28]. Differences in treatment outcomes have also been recorded with a higher risk of treatment failure from CXL or corneal transplantation in the paediatric population compared to adults indicating important gaps in the current understanding [29–32].

### Rationale

The total cost of keratoconus in Australia has been estimated to be approximately AUD 44.7 million per year to patients and the wider community [33]. The public health impact of keratoconus is amplified by the fact that the disease manifests early in life, thereby affecting patients through their prime education and earning years. Identifying individuals who are most at risk of developing the disease and those who may progress more rapidly is necessary to minimise long-term visual disability, financial and social pressures, and reducing the public health burden of the disease.

Research groups across the world have individually collected cohorts of patients with KC in an effort to better understand the underlying molecular causes, clinical characteristics and treatment options of KC but these studies have had limited generalisability and reproducibility across different ethnicities. The increasing availability of large sample sizes with data from KC patients globally, provides an opportunity to test more hypotheses with greater power and eventually potentially develop strategies that may allow for earlier detection and more appropriate treatment algorithms. For example, a recent study involving a number of international groups allowed for analysis of 4,669 KC and 116,547 control samples to identify for the first time 36 genetic loci in KC [34]. Thus, the benefits of large sample collections and collaboration are clearly apparent.

There are a large number of population-based studies internationally, but the insights from these studies into the risk and protective factors for keratoconus or ways to slow the progression have been inconsistent. Some of the problems confounding this research can be reduced by harmonising and pooling data across studies. KIC aims to harmonise data from international cohorts, in order to better understand the determinants of this condition.

The establishment of the global initiative of the KIC, is based on the tenet that Consortium Members have agreed to collect and share cross-sectional and longitudinal patient data to establish a single, large database. Consensus amongst founding members led to agreement that the data will be collected in a standard format across all sites so that data can be pooled or compared with ease across different study sites. The overall aims of the KIC are to: a) explore methods for early diagnosis, and staging of disease; b) identify clinical, genetic and other risk factors that contribute to keratoconus causation, progression and response to treatment amongst different populations and c) to establish longitudinal follow-up to analyse the effectiveness and variables influencing treatment outcomes. Combining clinical data with genetic and proteomic testing may further improve understanding of the relative contributions of genetic and environmental factors in the aetiology and progression of the disease and represents an ongoing goal of the consortium.

In addition to detailing KIC protocols, the baseline data of the KIC is presented in this paper.

## Methods

### Ethics and consent

The Study protocol was approved by the Royal Victorian Eye and Ear Hospital Human Research and Ethics Committee for all sites in Victoria, Australia (Project#10/954H) (Parts 1–3). The protocol was also approved by the Melbourne Health Human Research and Ethics Committee (Reference number: HREC/45365/MH-2018) to conduct the project at multiple centres across Australia for Part 1 and Part 2. This protocol follows the tenets of the Declaration of Helsinki and all privacy requirements were met. All non-Australian sites obtained their own approvals through their respective ethics/IRB committees, which includes consent to share data to facilitate collaborative projects.

A waiver of informed consent approved by Melbourne Health Human Research and Ethics Committee obtained (Reference number: HREC/45365/MH-2018) for the collection of clinical data in Part 1 as it consisted of standard clinical data obtained during a general eye consultation for patients diagnosed with and without corneal conditions. For the questionnaires in Part 2, verbal informed consent was obtained prior to their completion. This was also approved by the Melbourne Health Human Research and Ethics Committee (Reference number: HREC/45365/MH-2018) to conduct the project at multiple centres across Australia.

### Study design

The KIC is an international, multi-centre cohort study. It utilises a standardised data collection instrument (REDCap) to allow data from multiple patient cohorts from different centres to be collected in order to generate a large dataset, thereby increasing statistical power for analyses. The KIC members collect and share de-identified clinical data, response from questionnaires and tissue samples, if available. The data are collected using standard collection questionnaires that are common to all members. Thus, allowing for pooling or comparison of analyses between different sites. Statistical methodology required will vary dependent on the intended analysis and be incorporated as per best practice. Appropriate statistical methodology will be applied including chi square/ one way ANOVA and others for the analysis of demographic and clinical data, Rasch analysis for the quantitative traits and psychometric analyses and machine learning /artificial intelligence models for big data computational analyses.

### Participants

Data from all patients diagnosed with keratoconus by a clinician at a member site are entered into the database at each individual site. Patients of all ages are included, irrespective of any prior treatment they have undergone for keratoconus. Data from a second group of patients with no history of corneal disorders is collected as a control group. There are no exclusion criteria for patient collection aside from patients who have corneal disorders other than keratoconus.

### Study parameters

A Research Steering Committee had been established to finalise the key variables for data collection of the study. The main element was to capture the relevant information required in filling the knowledge gaps of keratoconus and at the same ensuring the data collection is not too onerous. To achieve these recent comprehensive studies such as Australian Study of Keratoconus were used as reference [20, 35–40]. Further KIC will continue to evolve with time allowing the new members to develop the current and accumulated data fields.

To regulate data collection among different institutions, the study parameters have been standarised, a detailed Standard Operating procedure has been designed for all the reseachers entering the data, data fields have been validated to accept the set range of values, data collection team is adequately trained prior to entering the data and researcher in charge of the site will undertake routine data collection checks.

The current data collection for this study can be divided into three parts. In Part 1, the data consists of standard clinical information including demographic data, ethnicity, ocular history, medical history (including a history of allergic disorders and eye rubbing), family history, biometrics, spectacle and contact lens prescriptions, visual acuity, clinical eye signs, imaging results, treatments performed and treatment-related complications. Data is recorded at baseline and follow up visits (Table 1).

**Table 1:** Study collection protocol at each visit

Assessment/Procedure	Baseline	Follow up visit
**Demographic data**	**X**	
**Medical History questionnaire**	**X**	**X**
**Disease status questionnaire**	**X**	**X**
**Quality of life questionnaire (IVI/IVIC)**^a^	**X**	**X**
**Economic burden questionnaire**^a^	**X**	**X**
**Clinical data**	**X**	**X**

^a^collected annually

As there is no globally accepted definition for defining the diagnosis and progression of KC, clinically diagnosed KC were considered as cases and follow up data is being obtained. One of the aims of this consortium is to establish gold standard diagnostic and follow up definitions for KC.

In Part 2, an economic burden questionnaire and a vision-related quality of life questionnaires (Impact of Vision Impairment (IVI) for adults and IVIC for children are administered for consenting participants at baseline and will be collected annually. Questionnaires were previously validated by Rasch analysis and have been published prior [41, 42].

In Part 3, tissue samples, where available, will include the collection of blood, saliva, corneal tissue and tears for subsequent analysis to investigate genetic and protein markers.

Additional sub-studies to investigate clinician and or patient perspective, for example treatment decision tree or social participation questionnaires, may be integrated over time following appropriate investigator and human research ethics committee approval.

### Data storage and security

The data management and collection tool, REDCap (Research Electronic Data Capture, https://www.project-redcap.org/) has been used to produce a unique database for the collection of the study variables. REDCap presents as a secure, web-based application designed to support data capture for research studies, providing an intuitive interface for validated data capture, audit trails for tracking data manipulation and export procedures, automated export procedures for seamless data downloads to common statistical packages and procedures for data integration and interoperability with external sources [16, 43]. REDCap provides a number of key advantages including in-built security features, real-time data validation and the ability for multiple sites and institutions to share data in real time. Data export functions are available to common statistical packages tools for offline analysis.

De-identified data is entered by each participating site into REDCap, hosted on the Centre for Eye Research Australia’s (CERA, Melbourne, Australia) domain. Each institution or site is provided with a site identification code and each participant given a numeric identifier in REDCap. Each member site retains their own unique identification codes to allow re-identification for input of longitudinal data. Members’ data remain the property of that member only and is only available to the contributing site as well as the data manager. While the baseline data are presented here, it is intended that further studies will be conducted between any number (or all) consortium members who wish to collaborate for any specific research question on KC. The use of REDCap also enables members to use their own data for auditing purposes. The REDCap system keeps an audit log, in real time, of any changes made to the data, including who accessed the data, the date, time and list of entries/ changes. Secure data transmission in encrypted format to the database (128-bit SSL via HTTPS) encrypted to a web-based server has been undertaken where each participant has been provided with a unique identifier generated by the system.

## Research direction

KIC will be a first worldwide study in KC that will allow us to undertake a unified analysis to enable better diagnosis of individuals at early risk of disease, explore a therapeutic algorithm specific to the pediatric KC, coming up with a revised classification system of KC useful for clinical decision making, identification of biomarkers to be targeted for future therapy, and finally drawing together of multiple sites for when there is a treatment ready for trialing and potentially avoiding graft surgery. The main aims of KIC are:

1. Early detection and assessing progression of keratoconus by identifying novel determinants.
2. Follow patients over time to gain an understanding of the effectiveness of therapeutic treatments on disease progression.
3. Develop genetic and laboratory investigations to supplement clinical findings.

## Results

### First project

The aim of this project is to provide baseline information about the current KIC members, demographic data, sample representativeness and their clinical features.

**Fig. 1:**
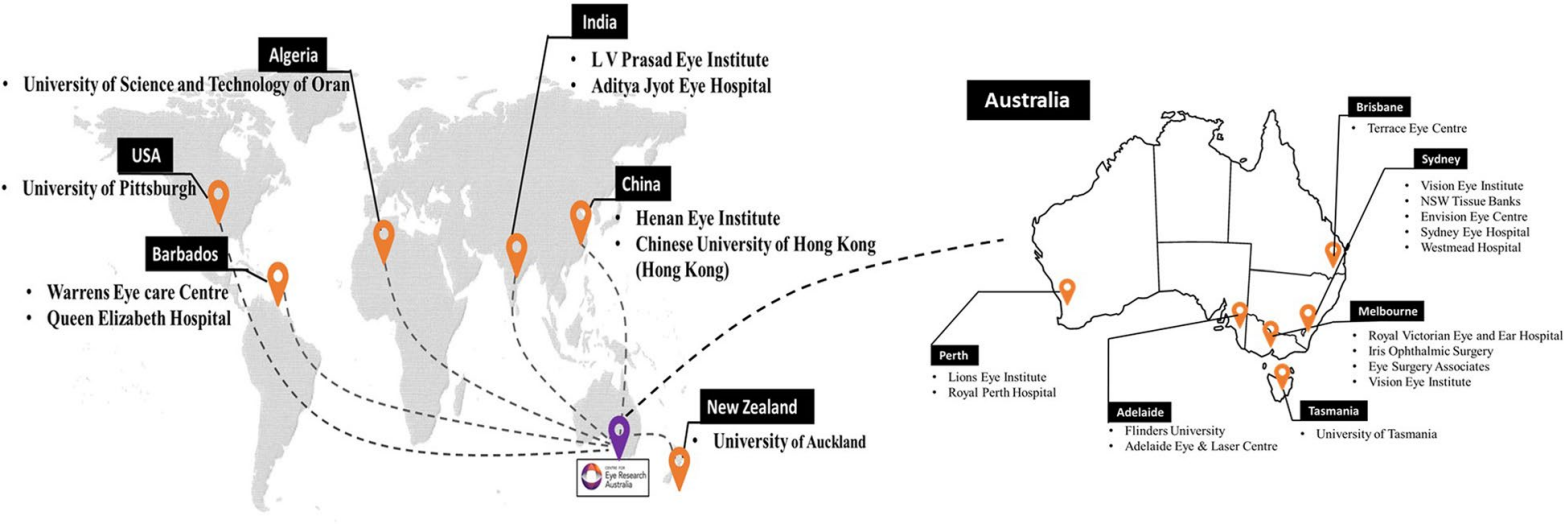
Map showing the current KIC members

Currently 40 Australian and international clinicians, clinics and/or hospital departments have joined the KIC (Fig. 1). These sites include Australia (multiple sites across Melbourne, Sydney and Perth), New Zealand (University of Auckland), India (Aditya Jyot Eye Hospital), China (Henan Eye Institute, Nanchang Eye Hospital, Chinese University of Hong Kong (Hong Kong), Algeria (USTOMB) and USA (Warrens Eye Care Centre).

Data on 2,220 individuals (1,030 keratoconus and 1,190 controls) from 15 sites has been entered into the REDcap database since 2018. The median age of keratoconus patients is 29.70 years (interquartile range 22.73–39.55). Primary variables represent routinely collected clinical data and therefore is vastly available across majority of the sites while missing data has been noted in questionnaire related fields. This is a potential limitation of our study and as such any such large, global consortiums. As keratoconus subjects return for their follow up visits, each site coordinators are encouraged to collect the missing data from the subjects during these visits.

**Fig. 2:**
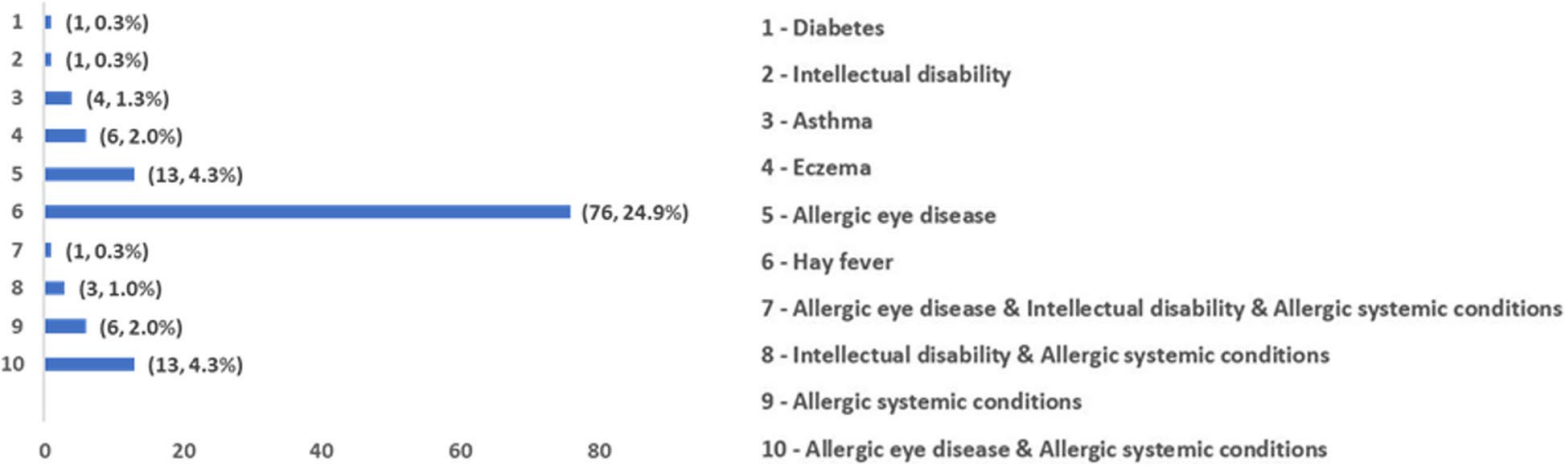
Systemic conditions of the keratoconus subjects recruited in KIC

Only 15.3% (*N* = 135; data available on 884 subjects) reported a positive family history of keratoconus while 57.7% (*N* = 488; data available on 846 subjects) self-reported a history of frequent eye rubbing. Ethnicity and gender data were available for 1030 and 1110 keratoconus patients, respectively. Of these, 454 (44%) were Europeans, 192 (19%) were Asians, 12 (1%) were Mixed (reported more than 1 ethnicity) and 96 (9%) reported their ethnicity as ‘unknown’ (Table 2). There were more males (672, 61%) than females overall, with the male preponderance also noted in each ethnic group. Self-reported history of systemic conditions was available on 305 keratoconus subjects. Of these, 181 (59.3%) subjects reported no systemic illness while the remaining had one or combination of conditions as shown in Fig. 2.

**Table 2:** Ethnic distribution of currently collected Keratoconus subjects in the KIC database

**Ethnicity**	**Keratoconus**	**Control**
	**Number (N) & Percentage %**	
Asian	192 (18.6)	(129, 11.0%)
SAARC	86 (8.3)	(207, 17.7%)
European	454 (44.1)	(204, 17.5%)
Hawaiian	12 (1.2)	(21, 1.8%)
Hispanic or Latino	2 (0.2)	(7, 0.6%)
African or African American	13 (1.3)	(9, 0.8%)
Middle Eastern	39 (3.8)	(36, 3.1%)
Indigenous/ Native origin	124 (12)	(323, 27.6%)
Unknown	96 (9.3)	(244, 20.9%)
Mixed	12 (1.2)	(10, 0.9%)

In the control group, a similar male preponderance was noticed (721, 61.2%) and majority of them belonging to Indigenous or Native origin (323, 27.6%) (Table 2). Asthma was the mostly self-reported systemic condition (89,8.3%), followed by eczema (81, 7.6%). In terms of eye diseases, Allergic eye disease was the commonly reported (58, 5.4%).

**Fig. 3:**
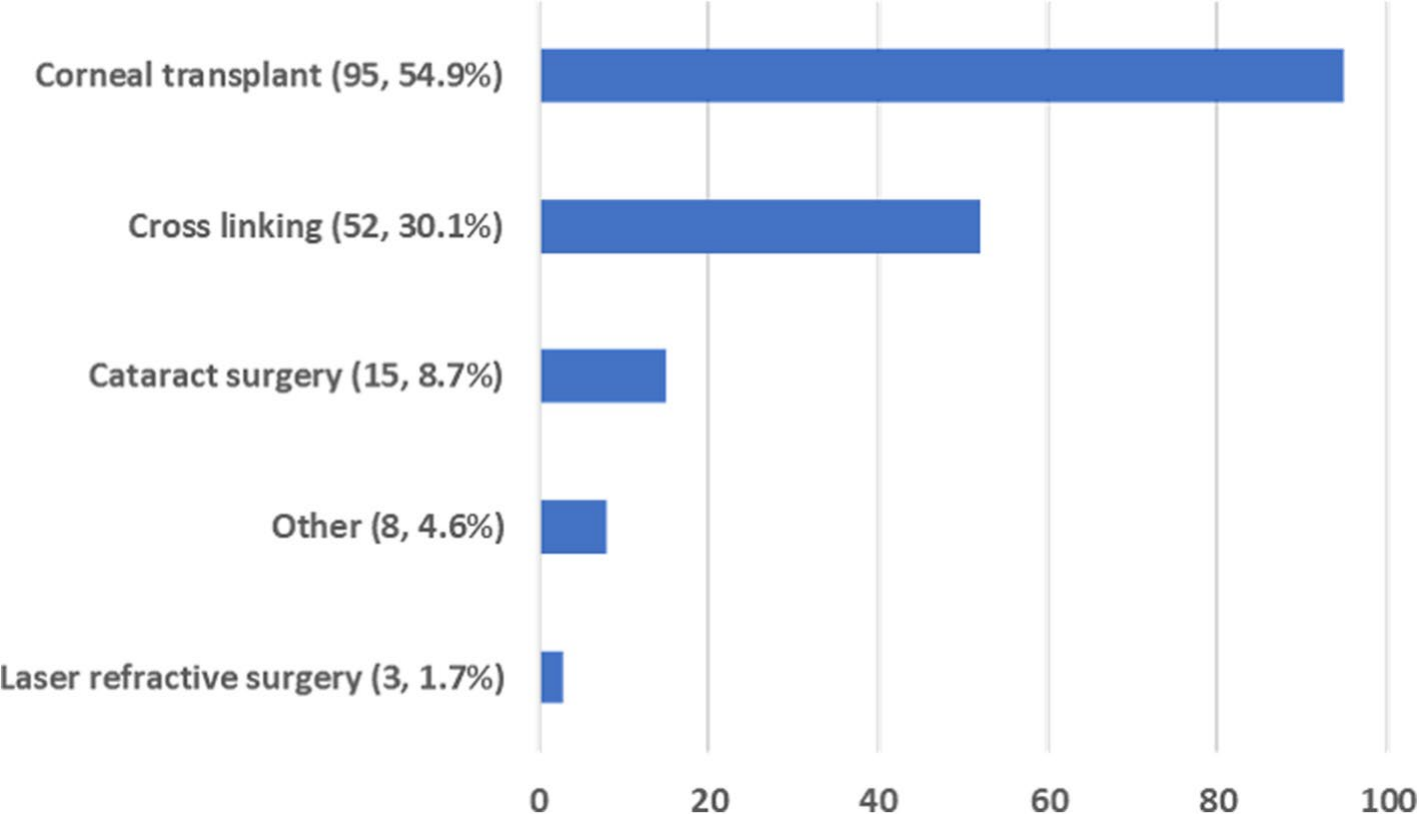
Prior eye surgery that the keratoconus subjects underwent

Corneal topography data included information relating to the corneal topography/tomography used and the various parameters commonly used by clinicians for the diagnosis and monitoring of keratoconus progression (Table 3). The Pentacam Imaging System was the most widely used corneal tomographer (50%), followed by Orbscan (32%), Casia (13%) and “others” (5%) (Wavelight, Atlas etc.). Figure 3 shows information on keratoconic eyes that had undergone some form of ophthalmic surgery which included cross linking, corneal transplant, cataract surgery or other.

**Table 3:** Baseline Corneal Imaging Data of keratoconus subjects in KIC

Corneal Parameters	Mean ± SD
**K1_F** (D)	46.43 ± 6.45
**K2_F** (D)	49.60 ± 13.40
**Km_F** (D)	48.81 ± 6.99
**BFS** (D)	7.27 ± 0.69
**K1_B** (D)	-6.93 ± 1.19
**K2_B** (D)	-7.68 ± 1.40
**Km_B** (D)	-7.20 ± 2.48
**Central corneal thickness** (µm)	477.96 ± 71.15
**Corneal thickness at the apex** (µm)	472.50 ± 68.12
**Corneal thickness at the thinnest point** (µm)	457.57 ± 64.55
**Location of the thinnest point in x axis** (mm)	0.03 ± 0.69
**Location of the thinnest point in y axis** (mm)	-0.52 ± 0.47
**Corneal volume** (mm3)	58.19 ± 4.65
**Chamber volume** (mm3)	188.40 ± 38.63
**ACD** (mm)	3.46 ± 0.44

*K1_F* Flattest keratometry reading of front cornea, *K2_F* Steepest keratometry reading of front cornea, *Km_F* Average keratometry reading of front cornea, *BFS* Anterior best-fit sphere, *K1_B* Flattest keratometry reading of back cornea, *K2_B* Steepest keratometry reading of back cornea, *Km_B* Average keratometry reading of back cornea, *ACD* Anterior Chamber Depth

### Future projects

A number of future projects utilising KIC data are currently planned, and aim to make comparisons across KIC cohorts, countries and ethnic groups of:

1. Geographic differences in clinical presentation of Keratoconus subjects
2. Comparison of risk factors for Keratoconus between adults and children
3. KIC Crosslinking Data–- Details on different protocols used, age, clinical factors and progression data
4. Evaluation matrix to discriminate Keratoconus versus normal corneas using Artificial Intelligence
5. Identify factors that contribute to disease progression at an earlier stage
6. Development of a globally revised classification system for keratoconus

## Discussion

The preliminary findings of the KIC identified that 15.3% of patients had a reported family history of keratoconus which represents an increase over historical findings of 6–10% [21]. Although it may represent an increase in incidence, this value likely reflects both a growing understanding of the disease and clinician diagnostic capabilities. Over half (57.7%) of the KC patients reported some degree of eye-rubbing. Literature suggests rates between 44–100% of patients reporting eye-rubbing with a recent meta-analysis indicating a threefold odds ratio of developing KC in eye rubbers compared to non-eye rubbers [44, 45]. Although eye-rubbing represents a significant risk factor, the variance within the literature and available populations, suggests that an incomplete understanding of the impact of eye rubbing on aspects such as disease progression. Combined, these outcomes therefore suggest a role for developing a larger international, longitudinal database to help further define clinical and demographic variables that may contribute to disease diagnosis and progression. Although clinical evaluation continues to be variable across cohorts, the presence of a single, standardised, large dataset should help refine data collection and provide an increasingly relevant platform to allow for more accurate and robust determination of risk factors for the disease, with the added provision of offering the ability to validate findings with other groups within the consortium. Furthermore, longitudinal data will allow analysis of risk factors for disease progression and assessment of treatment efficacy.

There have previously been two main longitudinal studies investigating KC. The Collaborative Longitudinal Evaluation of Keratoconus (CLEK) study recorded a number of variables amongst 1209 patients including high and low contrast visual acuity, corneal curvature, biomicroscopy and quality of life [46]. Results from this study significantly expanded clinician’s understanding of the natural history of the disease, including the possible role of gender and family history in progression of the disease and its effect on quality of life [47–52]. The Dundee University Scottish Keratoconus study (DUSKS) examined 200 patients at time of enrolment and after a mean of 1004 days. DUSKS provided valuable information regarding demographic and environmental associations with the disease including the role of contact lenses in KC, the role of eye rubbing and impact of scarring on disease progression [53, 54]. Significantly, DUSKS was the first longitudinal trial on KC to utilise corneal topography throughout the duration of the study.

The potential advantage of the KIC compared to CLEK and DUSKS is its international, multi-centre cohort design which allows examination of a wider range of KC parameters and the impact of ethnicity and differing environment factors on the disease. With ongoing recruitment of sites and patient data entry, the sample size has the potential to rapidly grow.

There are a number of published indices and grading systems used for KC based on visual acuity, biomicroscopic or topographic/tomographic signs [55–59]. Numerous additional indices may be important, such as corneal aberrations, corneal volume, internal astigmatism and corneal biomechanics [2, 58, 60–64]. More recently, epithelial thickness has been proposed as a diagnostic tool to diagnose and classify KC patients [65, 66]. The availability of numerous indices and the absence of standard criteria for the diagnosis and classification of KC indicates that we are no closer to differentiating the earliest stages of KC from normal corneas which represents an essential goal for identifying at risk patients for progression and further for those patients undertaking potential corneal laser refractive surgery [67, 68]. Significantly however, it also reflects the wide range of morphology within the disease. Of special interest to the KIC will be studies on risk factors and grades of disease for KC and in particular their association with prognosis and the outcomes of therapeutic treatments. In the future, targeted treatments, orchestrated across multiple trial sites will allow monitoring of treatment outcomes and provide information on the impact of different risk factors. The use of a large centralised, consistent database can provide researchers with an excellent platform to use developing technologies such as artificial intelligence to explore a variety of novel interactions within the data which may help resolve some of these knowledge gaps or help define future research directions.

The Sight Loss and Vision Priority Setting Partnership identified research priorities through consultation with patients, carers and clinicians [69]. Some of the top 11 priorities in corneal diseases can be addressed through the KIC projects: what is the cause of KC; how it can be prevented; what causes progression of KC; how can progression be prevented; development of new therapies such as genetic treatments for corneal disease; and how the outcomes of corneal transplantation can be improved.

The next phase of KIC is expanding the current database to include Optometrists, who are the primary eye care providers. As such some of the current Institutes do have Optometrists and Corneal Surgeons working together (example- L. V. Prasad Eye Institute, India), however we would like to expand to private practises where KC subjects will be separately monitored at majority of the disease stages. This includes at the beginning of the disease (for the diagnosis), in the middle of the disease for contact lens fitting and prescriptions of topical anti-inflammatories and antihistamine/mast-cell stabilizers, and at the end of the disease (often rehabilitating post graft corneas). Thus, recruitment continues to be ongoing and it is expected that follow up investigations from the KIC will bear the inclusion of optometrists appropriately. All investigators within the KIC are equally encouraged to present research ideas and opportunities.

We have recognised the need to expand KC research to a global level to overcome differences in study design, patient populations, sampling methods, outcome measurements and lengths of follow-up. Of note, the early members of the KIC represent a combination of ophthalmic and tertiary research institutions. This may provide a bias towards more significant, progressive cases. Given the increasing presence of topography and tomography diagnostic units in optometric practices, the KIC will expand to incorporate optometry practices to further explore the identification of early detection of KC and which features are associated with disease onset. Understanding the practice and clinical differences across allied health groups is likely to further increase understanding and awareness of KC protocols to the benefit of all patients. To assist the development of the KIC, revise the available data and understand future directions, it is intended for the KIC to meet at regular intervals. We believe this will limit concerns around data collection and further strengthen collaboration and developmental opportunities for all members.

The KIC is a powerful potential tool for groups to study many aspects of KC which will have important clinical implications when interpreting studies from different regions around the world. Its main strength is that it is led by its members, where all members have an equal opportunity to contribute not only to the data but lead individual projects of interest. An understanding of disease presentation and progression patterns among various ethnic groups is warranted and could be achieved through this global collaborative initiative. The KIC establishes a database with the aim of establishing the world’s leading consortium for this disease.

## Conclusion

KIC, a multi-pronged, multi-ethnic and multi-centre approach that constitutes a large-scale, international collaboration to undertake unified data analysis to enable a better understanding of KC. Ultimately, this will optimise diagnostic and treatment interventions for our patients.

## Data Availability

The datasets used and/or analysed during the current study available from the corresponding author on reasonable request.

## Abbreviations

KIC: Keratoconus International Consortium
CERA: Centre for Eye Research Australia
CLEK: Collaborative Longitudinal Evaluation of Keratoconus
CXL: Corneal cross linking
DUSKS: Dundee University Scottish Keratoconus Study
IVI: Impact of Vision Impairment
SAARC: South Asian Association for Regional Cooperation including Afghanistan, Bangladesh, Bhutan, India, Nepal, the Maldives, Pakistan and Sri Lanka
